# Spatial distribution and deployment of community–based distributors implementing integrated community case management (iCCM): Geographic information system (GIS) mapping study in three South Sudan states

**DOI:** 10.7189/jogh.04.020402

**Published:** 2014-12

**Authors:** Abigail Pratt, Martin Dale, Elena Olivi, Jane Miller

**Affiliations:** Population Services International, Washington DC, USA

## Abstract

**Aim:**

In late 2012 and in conjunction with South Sudan’s Ministry of Health – National Malaria Control Program, PSI (Population Services International) conducted a comprehensive mapping exercise to assess geographical coverage of its integrated community case management (iCCM) program and consider scope for expansion. The operational research was designed to provide evidence and support for low–cost mapping and monitoring systems, demonstrating the use of technology to enhance the quality of programming and to allow for the improved allocation of resources through appropriate and need–based deployment of community–based distributors (CBDs).

**Methods:**

The survey took place over the course of three months and program staff gathered GPS (global positioning system) data, along with demographic data, for over 1200 CBDs and 111 CBD supervisors operating in six counties in South Sudan. Data was collated, cleaned and quality assured, input into an Excel database, and subsequently uploaded to geographic information system (GIS) for spatial analysis and map production.

**Results:**

The mapping results showed that over three–quarters of CBDs were deployed within a five kilometer radius of a health facility or another CBD, contrary to program planning and design. Other characteristics of the CBD and CBD supervisor profiles (age, gender, literacy) were more closely matched with other regional programs.

**Conclusions:**

The results of this mapping exercise provided a valuable insight into the contradictions found between a program “deployment plan” and the realities observed during field implementation. It also highlighted an important need for program implementers and national–level strategy makers to consider the natural and community–driven diffusion of CBDs, and take into consideration the strength of the local health facilities when developing a deployment plan.

## South Sudan situation analysis

South Sudan has one of the highest childhood mortality rates in the world, with an infant mortality rate of 105 per 1000 live births [1]. A large quantity of these deaths can be attributed to malaria, which is endemic in the region [1]. In addition, the 2010 South Sudan Household Survey found that two weeks prior to the survey, one third of children had been ill with fever, 34% had suffered from diarrhea, and 19% had suspected pneumonia [1].

Prevention and treatment of these illnesses is challenging. In 2010, only 6.3% of children under five received all recommended vaccines, and only about half of children under five with diarrhea received oral rehydration therapy or increase fluids with continued feeding [1]. In 2010, only 12.4% of children under five with malaria were treated with artemisinin–based combination therapy (ACT) [1]. Furthermore, many patients receive ineffective medicines and treatments that are not in line with national policy [1].

Protracted civil war in South Sudan has significantly reduced access to services and is an ongoing challenge. It is estimated that only half of the population has access to health care services, with most living more than 5 km from the nearest facility [1]. Transport is scarce, and in the rainy season, roads are often washed out, leaving areas of the country completely inaccessible for months of the year. Where health facilities do exist, the quality of services has been low due to frequent stock–outs of medicines, inadequate staffing and supervision, as well as a lack of appropriate equipment and supplies. Currently, literacy rates are very low (only 13.4% among women and 35.4% among men) [1] and have led to a shortage of trained professionals in all sectors, including health.

In February of 2009, the Ministry of Health and international partners developed the ‘Implementation Guide for Community–Based Management of Malaria, Pneumonia and Diarrhoea – A Community Child Survival Program’. Integrated Community Case Management aims to dramatically improve the quality of the South Sudan community health program, through the mechanisms of improved case management of patients with fever, increased trust in both anti–malarials and community service providers as a result of official Ministry of Health endorsement, strengthening of links between communities and facilities, and reduction in unnecessary use of anti–malarials. Despite the challenges of implementation in a post–conflict setting, South Sudan is capable of upholding the same high quality standards for fever case management found in other malaria endemic countries.

At the heart of any iCCM program is the community health worker, referred to as community–based distributors (CBD) in the South Sudanese context. CBDs are community–based volunteer health workers, nominated by their communities and incentivized through non–monetary channels locally and from implementing organizations. There is no minimum educational requirement, as the national literacy rate is extremely low, though all CBDs are required to attend a five–day training course on the key skills (identification of danger signs, classification of malaria, pneumonia and diarrhoea, treatment of the three diseases and prompt referral) [2]. General global guidance recommends a network of CBDs at a ratio of 20–25 CBDs to one supervisor, 1 CBD to roughly 40 households (approximately six people per household equals one CBD per 240 people) [3].

This cadre of worker has operated in South Sudan since 2006 and is relied upon not only in iCCM but also in other strategy documents such as the Home Health Promoters policy. The CBD work force in South Sudan at the time of mapping, was roughly 13 000 strong [4], across eight states with Global Fund support and guidance from implementing agencies including PSI, Malaria Consortium, Save the Children, International Rescue Committee, Catholic Diocese of Torit and the Bangladeshi organization, BRAC.

There is an important link between the community and the public health facilities that must be strengthened and fostered when operating an iCCM intervention. Community ownership and engagement with the CBD will not only improve the quality of treatment through improved feedback mechanisms and quality of care oversight, but can also help lower attrition rate [5]. The facilities need to manage and supervise CBDs within their catchment area to ensure the movement of both commodities and data, in order to avoid gaps in service. As evidenced by the WHO, “*comprehensive CHW subsystems can be deployed across sub–Saharan Africa at a cost that is modest compared with the projected costs of the primary–health–care system. Given their documented successes, they offer a strong complement to facility–based care in rural African settings.*” [6].

This paper describes the trial of a low–cost mapping and monitoring system, not currently reliant on mobile health or high–tech solutions, though it is the first step in the direction of more complex mobile Health (mHealth) interventions, incorporating District Health Information System (DHIS2) or similar data warehousing software and analytic tools. A similar survey was conducted in Malawi to map their Health Surveillance Assistants [7], supporting the method and findings described here. This mapping exercise indicates that this type of program could be replicated in other parts of the world to aid in the appropriate and equitable distribution of health services, particularly at the community level, and could be used as a program management tool to monitor for continuous improvement, program coverage, and impact.

The mapping exercise was conducted with five main goals in mind. The primary goal was to assess geographical coverage of the PSI’s iCCM program and consider scope for expansion. This would enable implementers to conduct a gap analysis based on both quantitative data and visually mapped points. Second, the pilot would assess the extent to which the iCCM program complements health facility service provision (ie, positioning of CBDs in relation to facilities). In South Sudan, iCCM exists for communities in remote areas without easy access to health facilities, and it is extremely important not to create parallel systems and duplicate the public health service delivery. Third, the exercise would produce a physical map to assess the CBD supervision from a spatial perspective, focusing on distances covered by supervisors during support visits, drug supply and data collection. Fourth, the mapping would enable spatial analysis of program data, and potentially allow for the linking of the ministry DHIS with community–based geographic information system (GIS) maps. The fifth and final goal of the exercise was to gather important base data on existing CBDs and CBD supervisors (CBDS) to inform the recruitment of new CBDs and potential redeployment of existing providers – including information about age, gender and education. Save the Children conducted a thorough endline mortality survey in South Sudan which included significant demographic data but did not include mapping as a program management tool.

## METHODS

### Mapping design

In late 2012, PSI used GPS units and GIS software to map the location of 1275 CBDS and 111 CBD Supervisors, trained to conduct classification, treatment and referral services, in their communities. 119 health facilities in these areas were also mapped. 18 PSI iCCM Field Officers (FOs) were trained on the use of the data collection forms and GPS units and were responsible for the mapping of programming in six counties – Yei, Lainya, Morobo, Mundri East, Mundri West, and Wau. Field data was collected over the course of three months and because the exercise was initially designed for programmatic use, all CBDs/CBD supervisors were mapped (no sampling frame required). The only requisite inclusion criteria were that the CBDs had been trained by PSI during the life of the project.

A simple data collection form was developed to capture base data on CBDs and CBD supervisors (age, gender, education/literacy and GPS coordinates). Health facilities operating within the iCCM program were also mapped. The aim was not to measure program effectiveness or impact, but rather to paint an accurate picture of the geographical scope of the program, a foundation upon which to overlay program data.

FO’s were trained in the use of GPS devices, the data collection tool, and appropriate engagement techniques with CBDs during the mapping exercise. FO’s were expected to move to the CBDs, in order to alleviate any observation bias, and so that CBDs were not expected to alter their regular schedules.

Each Field Officer was assigned the task of mapping CBDs, CBD supervisors, and health facilities within their catchment area (mapping activity combined with supervision activity to minimise costs). Once data points and demographic information had been captured, FO’s were responsible for cleaning the data and exporting it to the monitoring and evaluation team. From there, the coordinates were compiled in the ArcSoft software and saved as a layer of points in .dxf format, which was then converted into a shape file that was superimposed over the map file. Data collected was quality assured, input into an Excel database, and subsequently uploaded to GIS for spatial analysis and map production.

Ethical approval was not required, however the Ministry of Health, via the National Malaria Control Program was involved in study design and rollout, as GPS mapping in South Sudan requires approval.

## RESULTS

### Key findings

**Figure 1:**
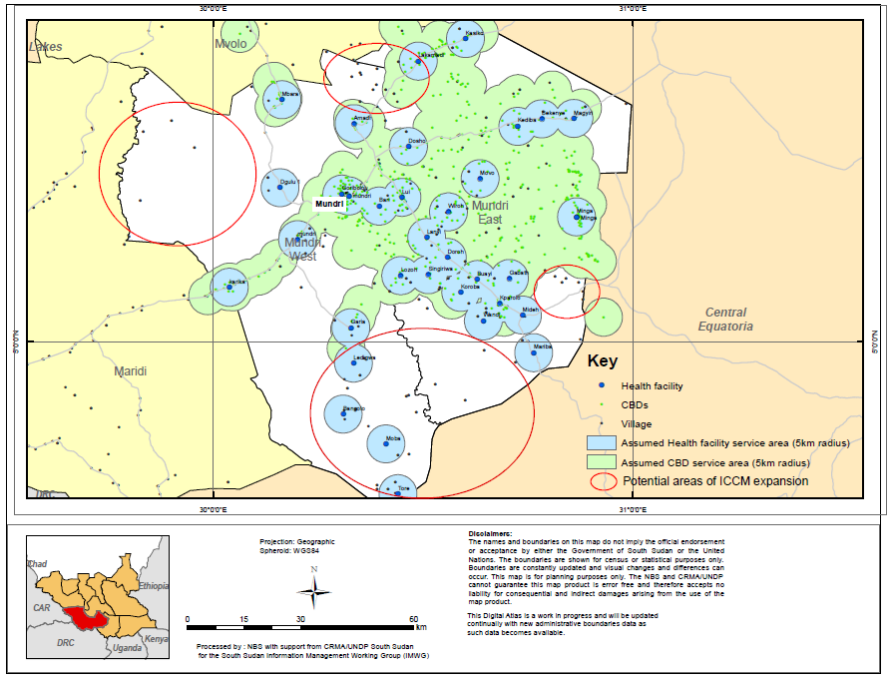
iCCM maping: Mundri East and West Counties, Western Equatoria.

**Figure 2:**
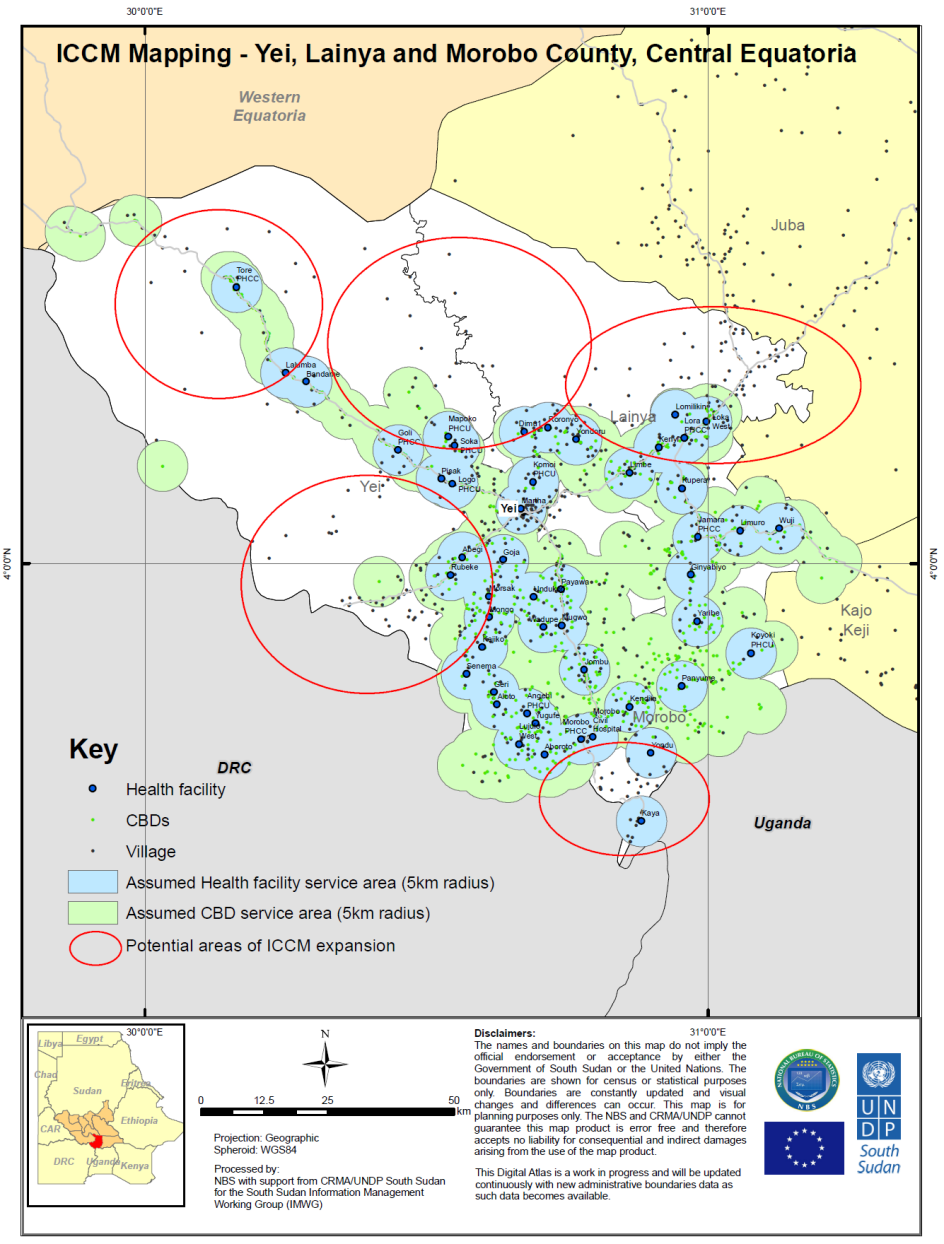
iCCM mapping: Yei, Morobo and Lainya Counties, Central Equatoria.

**Figure 3:**
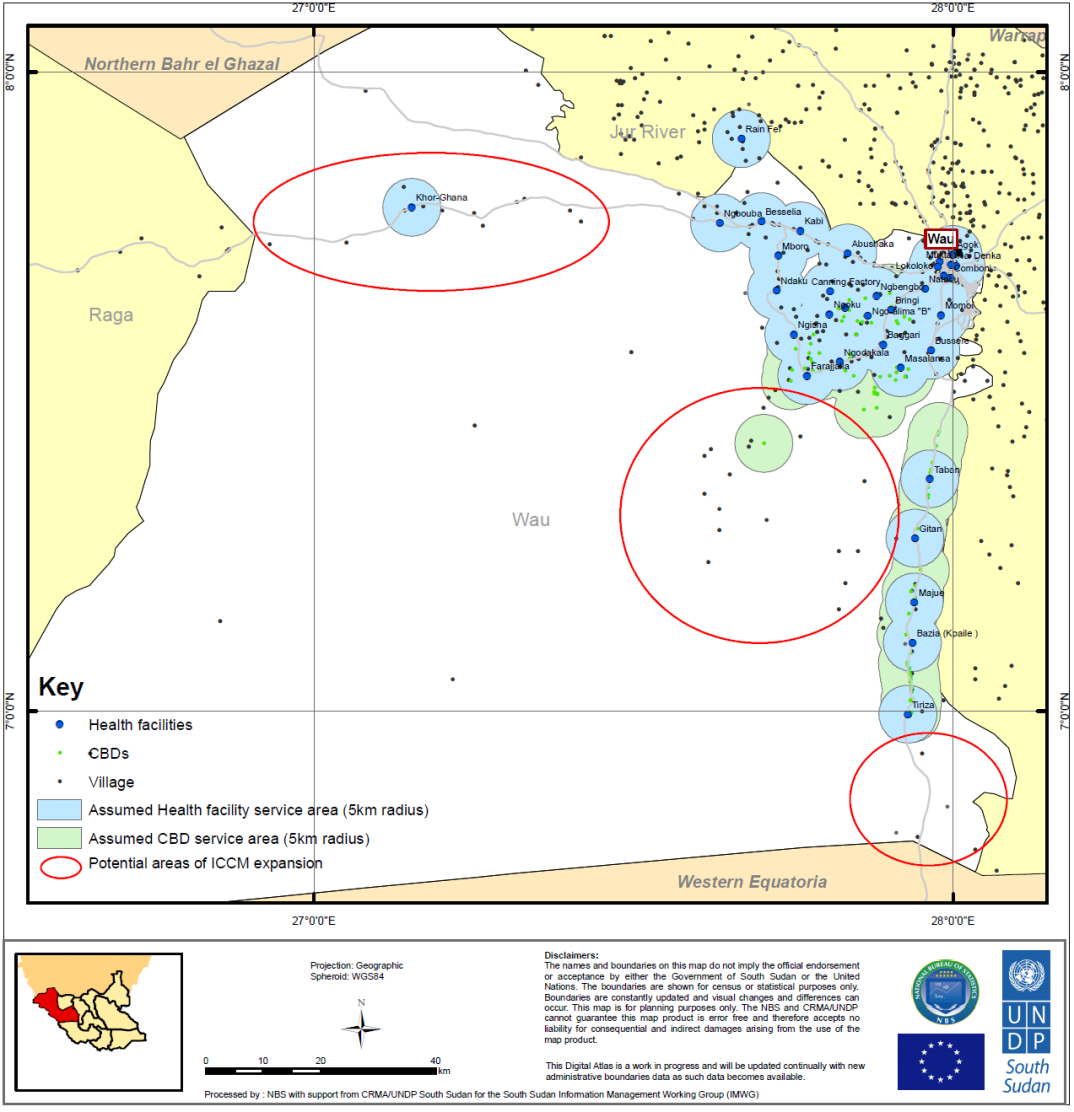
iCCM mapping: Wau County, Western Bahr el–Ghazal.

This exercise resulted in three county–level maps (**Figures 1**** to ****3**) pinpointing the positioning of CBDs and health facilities in project areas. The maps indicate CBD clustering near health facilities, where green and blue circles overlap. A significant proportion (69%) of CBDs are positioned within 5 km of health facilities. 79% in Western Bahr el–Ghazal (Wau); 58% in Western Equatoria (Mundri East and Mundri West); 77% in Central Equatoria (Yei, Morobo, and Lainya). The maps also highlight areas for potential expansion or reallocation of CBDs to areas designated as “underserved”.

A total of 1275 out of 1316 active CBDs were mapped between October and December of 2012 (**Table 1**). Mapping each CBD in their homes took longer than anticipated, due to standard challenges with roads and access, but 97% of CBDs were mapped along with all 111 active CBD supervisors, and all 119 referral facilities, 107 of which had a building and staff and were considered “operational”.

**Table 1:** Mapping of community–based distributors (CBD) quantities across three program areas

	Central Equatoria	Western Equatoria	Western Bahr el–Ghazal	Total
Number of CBDs mapped	646	539	90	1275
Number of CBDs not mapped	29	10	2	41
Total CBDs	675	549	92	1316
Number of mapped CBDs within 5 km of HFs	499	310	71	880
Assumed number of unmapped CBDs within 5 km of HFs	22	6	2	30
Total number of CBDs within 5 km of HFs	521	316	73	910
% of mapped CBDs within 5 km of HFs	77%	58%	79%	69%

HF – health facility

### Maps

Each map generated depicts one of the three states in which the program operates. Counties within the state not covered by PSI’s iCCM program are shaded in yellow, and bordering states are shaded in orange. The maps pinpoint CBDs and health facilities, surrounded by shaded 5 km catchment areas (green and blue respectively), and villages are similarly mapped. The maps indicate large swaths of the counties that are not currently covered by CBDs, demarcated in red. The mapping exercise also resulted in the identification of villages in the catchment area that were no longer occupied, which were recorded in the pilot findings but excluded from the maps.

**Figure 1**, with the map of Western Equatoria, shows comparatively complete coverage of inhabited areas, and less overlap of facility and CBD catchment areas. There are a number of villages in the northwest and southeast of Mundri West that should show increased CBD coverage. Figures 2 and 3 show a more clustered deployment of CBDs and areas of significant overlap, particularly in Western Bahr el–Ghazal.

### Gender distribution of CBDs and CBDSs

**Figure 4:**
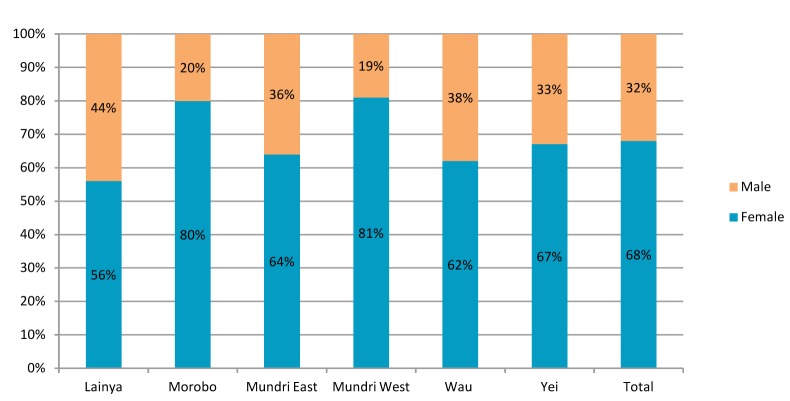
Ratio of male to female CBDs operating in the program areas.

**Figure 4** demonstrates that two thirds of the CBDs surveyed are women. This is in line with the standardized recommendations in South Sudan, as women are the primary caregivers and most often remain in or close to the home. There are a few areas, such as Lainya, where the gender distribution is more balanced.

**Figure 5:**
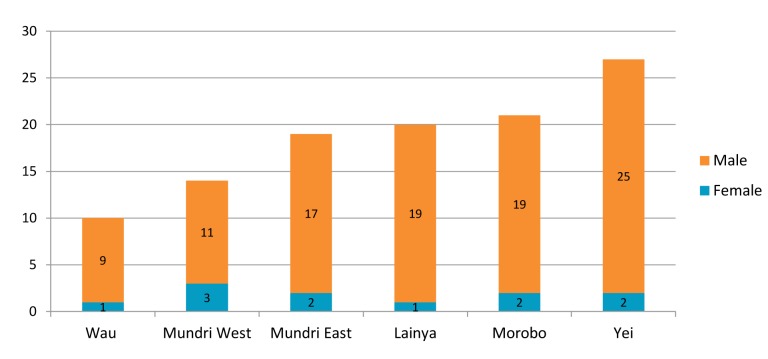
Ratio of male to female CBD supervisors operating in the project areas.

**Figure 5** shows that only 10% of CBD supervisors are female. The role of the CBD Supervisor includes a number of responsibilities beyond those required of the CBDs. supervisors are required to move long distances between CBDs for support supervision visits, either by foot, by bicycle and occasionally by motorbike. Supervisors are responsible for the quality assurance of CBD patient assessments and reporting, the latter requiring strong literacy skills. The supervisors are also salaried, in this particular instance, and it makes the position more competitive. There are also strong cultural considerations that impact this specific gender ratio. All of these different components seem to draw younger men into the position, though the feasibility of improving the gender balance should be explored prior to recruitment of new supervisors.

### Age, education and literacy

While there is no specific guidance for the optimal age cohort of CBDs, this study found that the average age is between 26–35 years, given that there is a higher rate of attrition in the younger cohort, and the older cohorts tend to be of lower literacy. This age range also corresponds with some of the more mature mothers, (ie, women who have had experience as caretakers or who have been trained in other health areas). Supervisors show a similar age profile.

While the focus of this research was spatial deployment of health workers, it is interesting to note that at least four out of five CBDs have been exposed to some level of education, higher than originally suspected. Training, assessment skills and recordkeeping have been tailored for low–literacy adults with minimal traditional education. Literacy rates are nearly 70% in five out of the six counties implementing iCCM activities. The low literacy rates reported in Wau have been flagged, as supervision visits should reflect increased support given that these CBDs may struggle to complete patient registers. Over three quarters of CBD supervisors have some degree of secondary education, as per the minimum requirement during recruitment. CBDs are occasionally promoted to supervisors without meeting this requirement, based on performance.

## DISCUSSION

The Results outlined the pilot findings, highlighting overlaps in programming and suggesting inefficiencies, but the essential next steps involve understanding how these factors impact program outcomes, and tailoring program design and implementation.

### Resulting management actions

In early 2013, PSI South Sudan took decisive management action in response to the high density of CBDs operating within the 5 km catchment area of public health facilities and other CBDs. The proposed response was a downsizing of CBDs in overlapping areas, from 69% to 49%, which freed up available funding to expand into underserved areas. Because the mapping research was aimed at improved CBD deployment, the results were used to make concrete management decisions to expand coverage through three principle steps.

The first step was the identification of superfluous CBDs for dismissal. This decision was based on a two–pronged strategy involving central–level analysis and a more nuanced performance–based field analysis. The centralized analysis included the use of an algorithm that took into account mapped CBD clustering, geographical overlap with operational health facilities and the caseloads of individual CBDs. This algorithm resulted in a list of CBDs for potential dismissal from the program. The field–based analysis took a closer look at facility service provision in catchment areas (for example hours of operation, continuity of supply, staffing capacity) as well as the capacity of CBDs to perform the required tasks and report in a timely manner. These ground–level considerations enabled the short–listing of low–performing CBDs for dismissal. The two lists were compared and a list of 72 CBDs was drawn up.

The second step was to hold sensitization meetings with communities that had excess CBDs, or CBDs that were clustered too close to the health facilities, to explain to them that those CBDs would be dismissed and that new CBDs would be selected in areas with greater need.

The final step in the reallocation and redistribution process was the dismissal of those 72 CBDs and the recruitment, training and deployment of 150 new CBDs (**Table 2**). The intention was to ensure more appropriate distance between independently operating CBDs and from the health facilities. These new CBDs were recruited based on county migration maps, highlighting occupied villages without essential health services.

**Table 2:** Redistribution of community–based distributors (CBDs) following mapping exercise

	Central Equatoria	Western Equatoria	Western Bahr el–Ghazal	Total
Total CBDs	675	549	92	1316
Proposed allocation of new CBDs	150	115	35	300
Total incl. new CBDs	825	664	127	1616
% of all CBDs within 5 km of HFs if new CBDs are outside HF coverage	60%	47%	56%	54%
Proposed reduction in CBDs within 5 km of HFs	72	30	10	112
% of all CBDs within 5 km of HFs	52%	42%	48%	48%

HF – health facility

### General analysis of findings

This mapping exercise and subsequent redeployment intervention raised several critical issues and challenges associated with planning and implementation.

***Proximity of CBDs to health facilities – complementing or competing?*** Standard iCCM programming encourages close supportive links between CBDs and their referral Health Facilities. In a mature system, CBDs are supported entirely by the corresponding Health Facility through training, re–supply and data exchange. In the fledgling South Sudanese system, non–governmental organizations have stepped in as needed, to bridge the two service provision levels. This mapping exercise has indicated that 69% of PSI’s CBDs were operating within the 5 km catchment area of a functional health facility (HF), clearly visible on the maps. Through the comparison of proximity to health facility, functionality of the facility itself (operational hours, staffing, and caseload data) and CBD caseload data, program managers can get a sense of the complementary or competitive nature of the CBDs.

Anecdotally, CBDs were reporting a high incidence of referral *from* Health facilities, known as ‘reverse referral’. For example, facilities were documenting cases of fever and referring caregivers to CBDs operating in the surrounding areas, as stocks of ACTs and RDTs at facilities were low. The phenomenon highlights the need for national level supply chain support and analysis, to identify breaks or bottlenecks in the system and generate solutions or means to fill this gap. CBDs are meant to extend the reach of the public health system, not replace the existing structures. Continued reliance on donor–funded community–based health providers also raises the important question of sustainability.

***Volunteer selection process.*** The CBD nomination or voluntary process also appears to contribute to the geographical clustering. While involved in the preparation and sensitization surrounding the CBD selection process, PSI Field Officers do not select, approve or disqualify CBDs that have been chosen or put forward by the community. According to the guidelines, CBDs are volunteers, endorsed by the community as respected, upstanding and primarily stationary members. Women are encouraged to participate as CBDs, though this is not always reflected in the CBD cadre.

Because the program is community–based and community–led, there is always uncertainty as to whether or not volunteer nomination will adhere to selection criteria. The findings here demonstrate that while the majority of personal characteristics match the desired profile, the physical location of these CBDs and their relative proximity to a health facility is not a disqualifying factor. This begs the question, is there a requirement to redeploy/reallocate CBDs, or is the need being adequately met, and the recommendations should include a greater degree of flexibility?

The visual mapping of CBDs indicates that coverage by community health programming is ultimately affected by local recruitment and selection/nomination proceedings. As recommended by Jacobs et al., “*examination of an individual or household’s ability to access services, and elements within the health sector or program that prevent uptake,* [allows for the development] *of context–appropriate improvements to the intervention and maximize coverage and uptake.*” [8]. This information about community access, paired with CBD/facility caseload data, would create a more thorough picture of the health system. Currently, it is difficult to know if the systems are supporting each other, dividing the work and providing triage, or if one or both sides are dysfunctional.

***Underserved communities – where are all the CBDs?*** The red circles on the maps indicate large swaths of each county that, at the time of mapping, were not supported by a CBD. This showcases a gap in program implementation and depending on the community needs, these villages should have been prioritized in the reallocation and subsequent round of CBD selection and training. This component of the exercise highlights the need for precise and comprehensive program planning, along with a strong coordinated effort from the county level health officials and the implementing partners. Improved coordination between these two groups can improve coverage of potentially underserved areas.

### Mapping limitations

Outdated maps of states and counties of South Sudan mean scant information about the “operational status” of primary health care units and centers (including referral hospitals, hospitals, primary health care centers and primary health care units) as well as real–time land use by populations. It is also possible that some of the more traditional nomadic communities were not included in the mapping exercise, though every attempt was made to include these mobile groups. Population distribution at the time of the exercise relied on information from the South Sudan National Bureau of Statistics. This meant that repositioning/redeployment of CBDs required additional time, surveying and coordination to ensure that they were being chosen in the areas of greatest need.

Lessons learned and study methods from such a deployment case study are practical and useful for others wishing to conduct similar operational research, but it must be noted that results and conclusions may not be widely generalizable.GPS mapping can and has worked in other contexts, but regulations and policies developed as a result need to be tailored to the area of operation.

### Challenges

The programmatic decision to reduce CBDs in some areas, and increase in other areas was highly sensitive, despite the clear inequity in access, imbalance in CBD density, redistribution of resources, and potential to improve the quality of programming. Re–deployment also meant the training of new CBDs, establishment of new relationships with health facilities and this may have been interpreted as a step backwards programmatically, despite improvements to the quality of the program.

The current political situation also presents challenges to the continuation of many public health programs, including iCCM. Although these community–based programs are the principle mechanism of reaching remote and underserved areas, they rely heavily on the national supply chain for commodity management, in addition to program implementing agencies for funding and monitoring. Internal displacement and high rates of attrition add to the challenges of this already complex program.

### Recommendations

***mHealth solutions.*** This initial mapping exercise was the first step in shifting towards a more advanced and practical data management system. Further down the line, as mobile network coverage improves across the country, it will be feasible to create a mobile health (mHealth) system that collects caseload data instantly from CBDs. This data can be sent to a centralized server by SMS and seamlessly uploaded into DHIS2, where constant monitoring can alert implementing teams, as well as national level decision makers, to make evidence–based policy and action plans.

The mapping of CBD and use of mapping technology fits in very nicely with other mHealth programming, particularly in the areas of data access and monitoring and compliance [9]. When paired with a Quality Assurance component, generated through Provider Assessments, it also has the potential to empower PSI Field Officers to monitor providers’ performance and allocate supportive supervision according to need as opposed to a generic schedule.

***Improved quality of programming through retention.*** Quality Assurance programming should be implemented and management decisions should be driven by caseload data and quality of care provided by the CBD. Program quality can also be improved by maintaining the strong individuals among the CBD and CBD Supervisor cadre through varying types of motivation. Young men are primarily CBD supervisors, and yet they often leave the cadre in search of higher paying work, to the detriment of the program.

## CONCLUSION

The results of this mapping exercise provided a valuable insight into the contradictions found between a program “deployment plan” and field implementation. It also highlights an important need for program implementers and national–level strategy makers to consider the natural and community–driven diffusion of CBDs, and take into consideration the strength of the local health facilities when establishing catchment areas.

The results from this mapping exercise demonstrate that the process can be employed as a low–cost management tool for the distribution and deployment of community health workers or health services, for many different disease areas in addition to malaria, as it simple to use, affordable and accessible through different global funding sources, and it can aid in both effective implementation and planning.

The system can improve allocation of scarce resources, and with additional technology and quality assurance measurements, it increases the ability to “keep” high performing CBDs and CBDs that are valued in the community for providing a quality service to their patients.

Additional research and funding will be required to go to scale in South Sudan, but there are organizations investing heavily in building the capacity of the community health worker, by equipping them appropriately, using adult training techniques and providing a network of supportive supervision and continuous training that will give CBDs the confidence to do their jobs well.
